# Factors influencing open gingival embrasures in orthodontic treatment: a retrospective clinical study

**DOI:** 10.1186/s40510-025-00554-6

**Published:** 2025-02-21

**Authors:** Erkang Tian, Kaihui Luo, Yimei Zhou, Fulin Jiang, Rongxiu Zhang, Lisa Liu, Hui Zhao, Jiawei Hong, Juan Li, Fangyuan Cheng

**Affiliations:** 1 Sichuan University, Chengdu, China; 2 Chengdu Wuhou Heyan Yuese Dental Clinic, Chengdu, China; 3 Department of Stomatology, Affiliated Drum Tower Hospital, Medical School of Nanjing University, Nanjing, China; 4 Chongqing University Three Gorges Hospital, Chongqing, China; 5 First Affiliated Hospital of Bengbu Medical University, Bengbu, China; 6 University of Electronic Science and Technology of China, Chengdu, China; 7 Chengdu Boltzmann Intelligence Technology Co., Ltd, Chengdu, 610095 China

**Keywords:** Orthodontic treatment, Open gingival embrasure space (OGES), Influencing factors, Retrospective analysis

## Abstract

**Objective:**

This study aims to evaluate the incidence of open gingival embrasures (OGES) after orthodontic treatment and analyze its correlation with various clinical and radiographic parameters.

**Methods:**

We retrospectively analyzed 330 orthodontic patients at West China Hospital of Stomatology from 2016 to 2023, categorizing them into Non-OGES (200) and OGES (130) groups based on post-treatment OGES presence in the central incisor area. Basic information of patients, pre- and post-treatment lateral cephalometric radiographs, and cone-beam computed tomography (CBCT) data were collected. Chi-square tests, two-sample t-tests, Welch’s t-tests, and Mann–Whitney U tests were used to compare differences in gender, initial age, treatment duration, and cephalometric and CBCT indicators between the two groups. Binary logistic regression analysis was further employed to explore the clinical characteristics and cephalometric indicators of the study population.

**Results:**

Univariate analysis revealed that the occurrence of maxillary central incisor OGES was significantly correlated with gender, initial age, treatment duration, and related cephalometric and CBCT indicators (*P* < 0.05). Similarly, the occurrence of mandibular central incisor OGES was also significantly associated with gender, initial age, treatment duration, and specific cephalometric changes (*P* < 0.05). Binary logistic regression analysis indicated that the occurrence of maxillary central incisor OGES was significantly related to initial age, treatment duration, and the change in the U1-SN angle, while the occurrence of OGES in the mandibular central incisor area was mainly related to initial age and treatment duration.

**Conclusion:**

Orthodontic treatment plans should consider a variety of influencing factors, including initial age, treatment duration, anterior tooth angle and position, root-bone relationship, and the distance from the anterior tooth contact point to the alveolar crest, to prevent or reduce the occurrence of OGES after orthodontic treatment, thereby improving patients’ aesthetic outcomes and periodontal health.

## Introduction

Open gingival embrasure space (OGES), also known as the "Black Triangle," refers to the visible triangular gap formed when the interdental gingival papilla cannot completely cover the gingival embrasure space [1]. The presence of OGES not only disrupts the harmonious aesthetic appeal during smiling but may also lead to food impaction [2], thereby affecting periodontal health [3, 4]. According to research reports, orthodontic treatment can contribute to the occurrence of OGES [5, 6], with statistics indicating a high incidence rate of 35.4–43.7% among orthodontic patients [7–9]. With the increasing pursuit of aesthetics and the growing number of orthodontic patients, the open gingival embrasure space in front teeth has garnered increasing attention.

Although previous studies have explored the influencing factors of OGES, there is still controversy regarding these factors, possibly due to differences in research sample size, inclusion and exclusion criteria, and measurement items. Most of studies focuses on gender [10, 11], age [8, 10, 12–14], gingival biotype [15], oral hygiene status [16], treatment duration [17], tooth extraction [18–21], and crowding [8, 22], but often ignores the consideration of tooth movement variation during treatment. In particular, there is a lack of reports utilizing cone beam computed tomography (CBCT) technology to conduct in-depth research on the influencing factors related to OGES.

The null hypothesis of this study is that there is no correlation between the occurrence of OGES in the central incisor area and factors such as gender, age, duration of treatment, method, tooth extraction, vertical and sagittal skeletal patterns, dentoalveolar height, inclination and movement of central incisors (*P* < 0.05, with a study power set at 0.8). Our objective is to test this hypothesis by examining the relationship between these factors and the development of OGES.

## Materials and methods

### Sample collection and grouping

This study has been approved by the Medical Ethics Committee of West China Hospital of Stomatology (Approval Number: WCHSIRB-D-2024-153), and followed the contents in the Declaration of Helsinki concerning human subjects. We retrospectively collected data from patients who completed treatment at the Orthodontics Department of West China Hospital of Stomatology, Sichuan University, between January 2016 and December 2023. The presence and location of Open Gingival Embrasures (OGES) were descripted in medical records. This study focuses on the analysis of OGES in the central incisor area, with specific inclusion and exclusion criteria as follows:

Inclusion criteria:

1. Patients in the permanent dentition stage;
2. Complete information, including intraoral digital photographs before and after orthodontic treatment (including frontal intraoral digital photographs), lateral cephalometric radiographs, and complete medical records;
3. All patients have received oral hygiene education.

Exclusion criteria:

1. The presence of OEGS in the central incisor region before treatment; OGES exists between the lateral incisors and canines, but not between the central incisors;
2. The presence of a gap between the maxillary and mandibular central incisors, or the presence of supernumerary teeth between the central incisors before treatment;
3. Congenital missing incisors or extraction of incisors during treatment;
4. Patients who underwent orthognathic treatment or received periodontal surgery during treatment;
5. Patients who underwent secondary orthodontic treatment;
6. Poor oral hygiene, severe gingival bleeding, acute hypertrophic gingivitis in the anterior tooth region, deep overbite, restorations in the central incisor region, or tooth crown defects involving tooth contact points, which affect the judgment of the gingival embrasure space region;
7. Systemic diseases, such as diabetes.

After screening, a total of 330 patients met the inclusion criteria. Patients with OGES in the central incisor area before treatment (31 people), those without OGES between the central incisors but with OGES in other areas (5 people), and other patients who did not meet the criteria were excluded, and finally, patients who met the requirements were included.

**Fig. 1:**
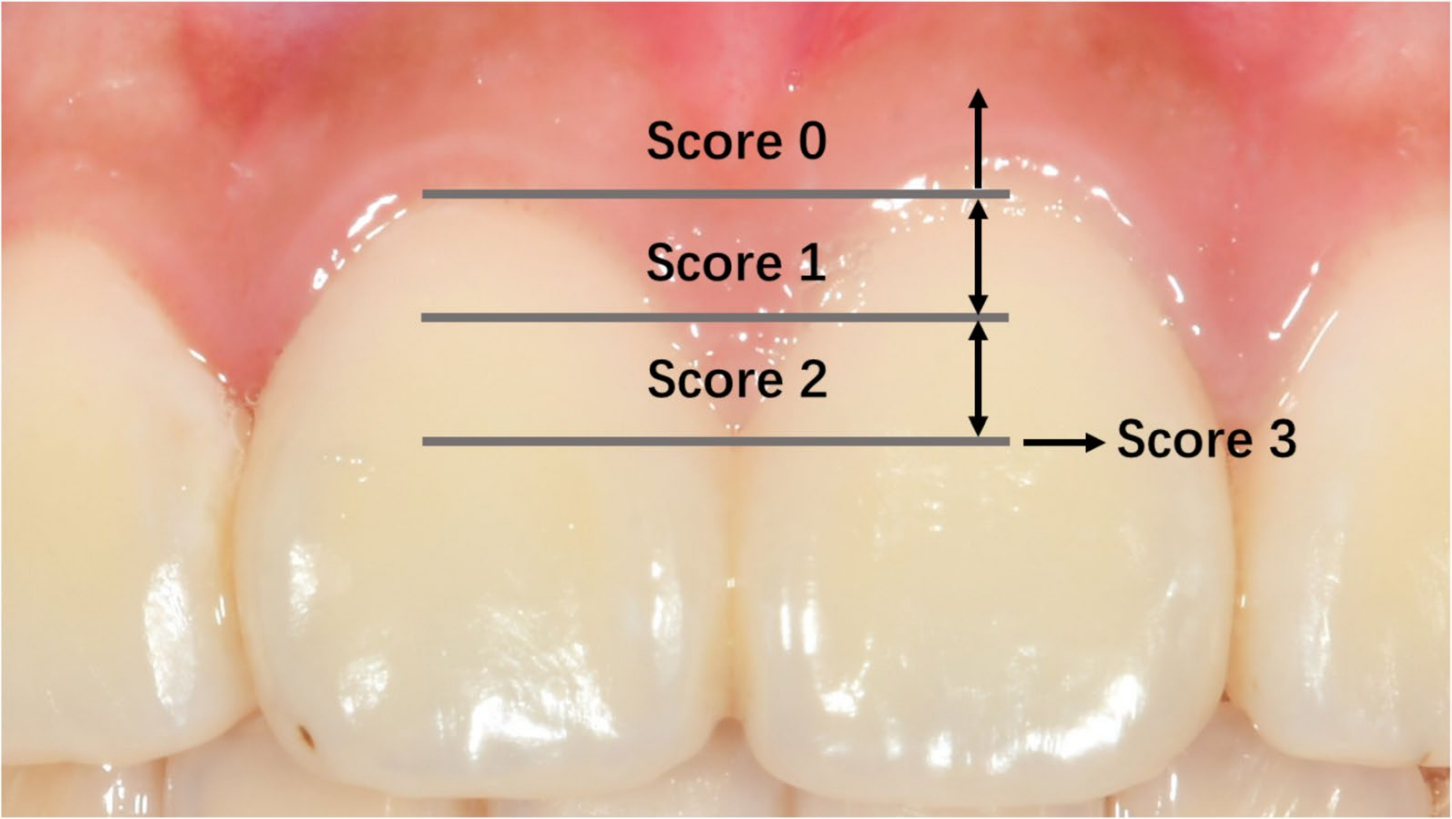
PFI Grading Schematic: PFI assesses the shape of the interdental papilla through intraoral digital photographs. This index uses the most convex point on the marginal gingival line and the contact point of the adjacent tooth as references, measuring the vertical distance between them as the gap height, and comparing it with half of the papilla height. Score 0: Severe, no interdental papilla, and no most convex point on the marginal gingival outline; Score 1: Moderate, the height of the interdental papilla is less than half of the gap height, and the most convex point on the marginal gingival outline exists; Score 2: Mild, the height of the interdental papilla exceeds half of the gap height but does not reach the most convex point, and is not coordinated with the shape of the adjacent papillae; Score 3: Normal, the interdental papilla fills the interdental space and is coordinated with the shape of the adjacent soft tissues, representing the most ideal soft tissue shape; Score 4: Hypertrophy of the interdental papilla

**Fig. 2:**
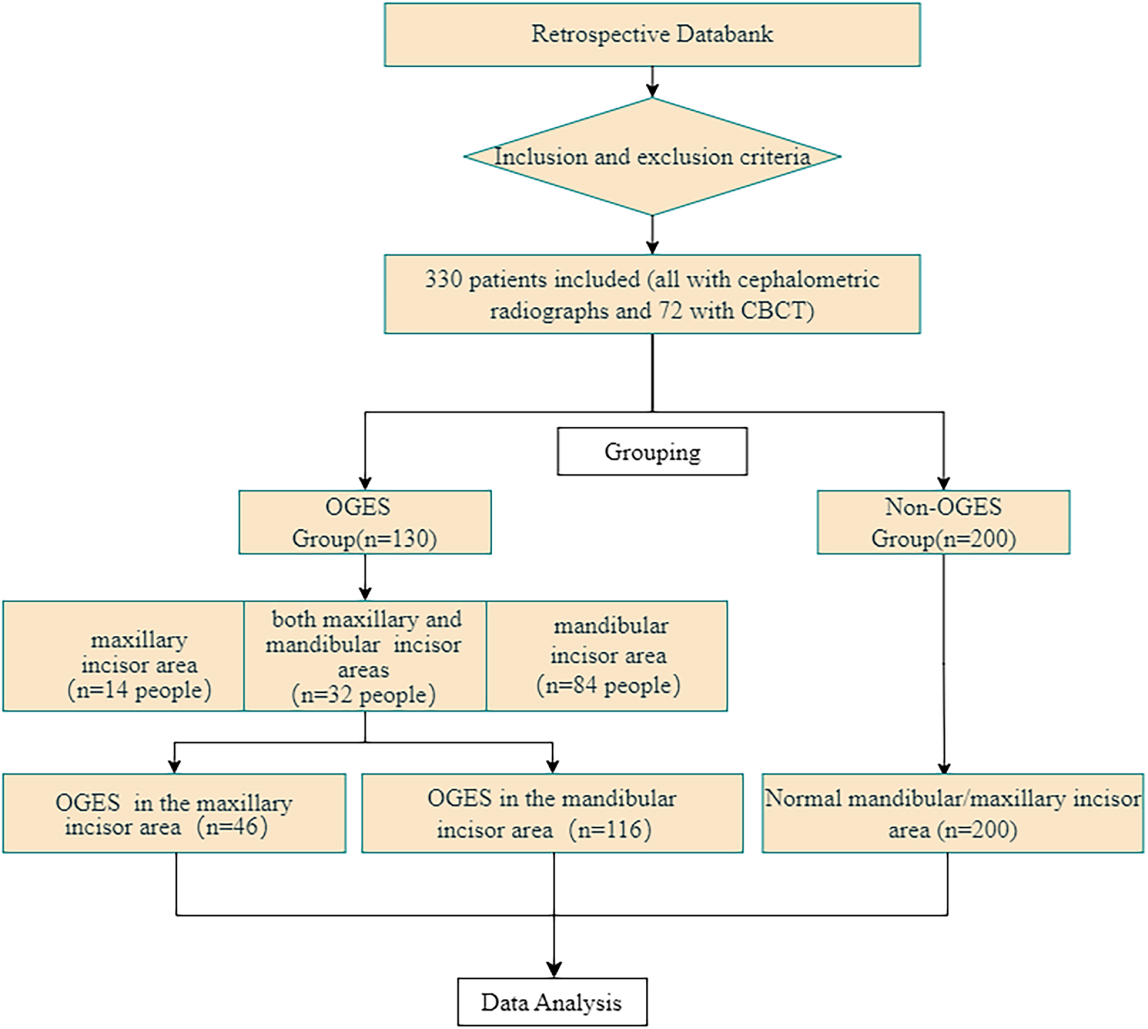
Experimental Flowchart

As shown in Fig. 1, in this study, dentists graded the shape of the gingival papilla in the central incisor area using the Papillary Fill Index (PFI) proposed by Jemt et al. [23] Among them grades 0, 1, and 2 were included in the OGES group, while those with grade 3 were included in the Non-OGES Group (those with grade 4 were excluded). Ultimately, the participants were divided into the OGES group (130 individuals who developed OGES in the central incisor region after treatment but had no OGES before treatment) and the non-OGES group (200 individuals who had no OGES in the central incisor region before and after treatment). Intraoral digital photographs and lateral cephalometric radiographs before and after orthodontic treatment were collected from all patients, and the images were saved in JPG format. Information such as gender, age at the first visit, duration of orthodontic treatment (accurate to the month), tooth extraction, and extraction site was recorded by a doctor. Additionally, for the OGES and non-OGES groups, CBCT data before and after orthodontic treatment were saved for 39 and 33 individuals, respectively. The flowchart of this experiment is shown in Fig. 2.

### Landmarking and measuring of lateral cephalometric radiographs

**Fig. 3:**
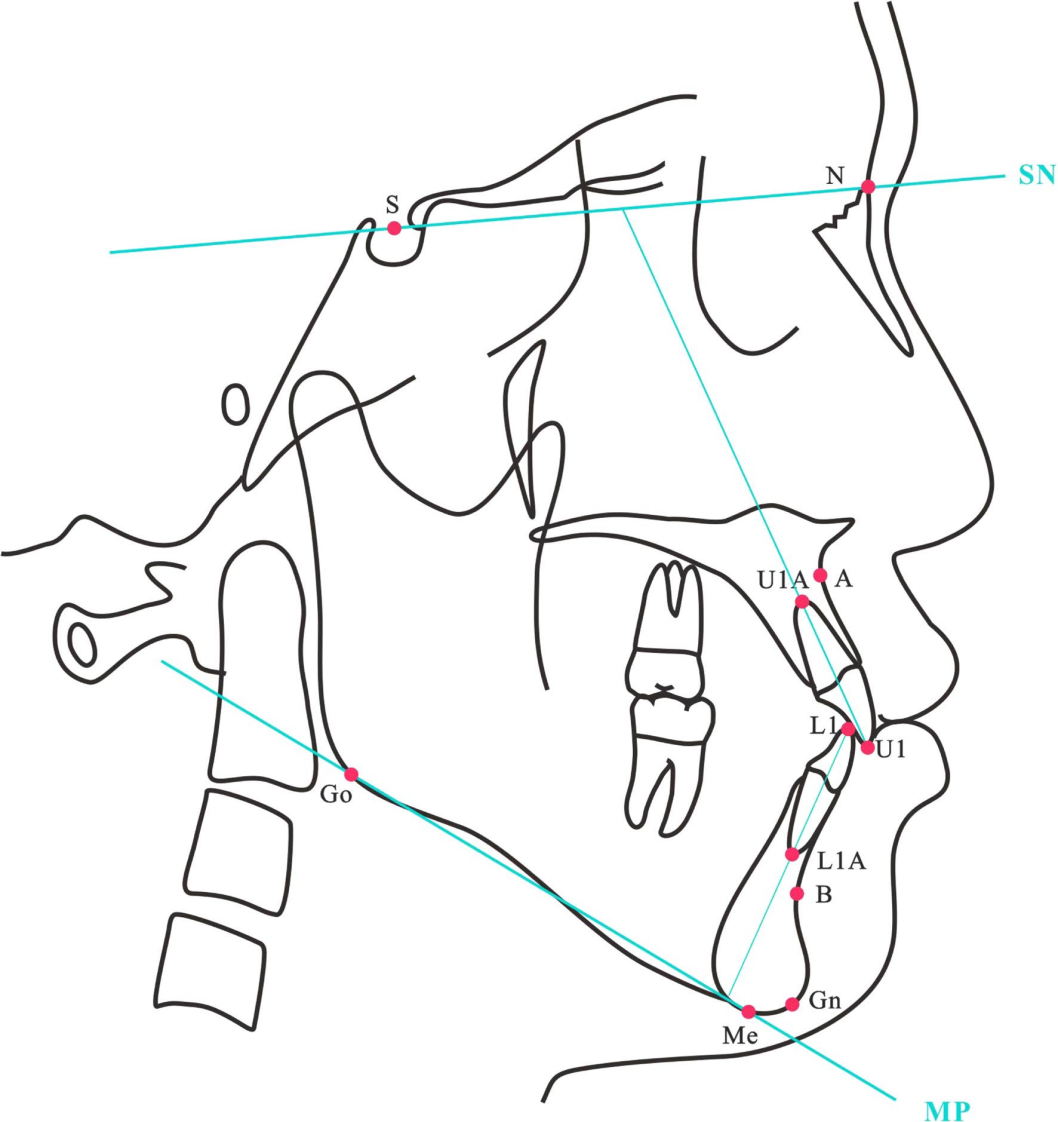
Landmarks used for cephalometric analysis. S: Sella; N: Nasal; Gn: Gnathion; Go: Gonion; A: Subspinale; B: Suprakental; UI: upper incisor; UIA: upper incisor apex; LI: lower incisor; LIA: lower incisor apex; MP: Mandibular Plane; SN: Anterior cranial base plane

A dentist finished cephalometric analysis by an platform [accessible at (https://www.zhibeicloud.com)]. The system has an average error as low as 0.94 ± 0.74 mm and an average accuracy rate of 89.33% [23]. The landmarks used for cephalometric analysis are shown in Fig. 3. T1 and T2 represent the measurement items before and after orthodontic treatment respectively. The treatment measurements before and after for U1-SN (°), U1-NA (mm), IMPA, and L1-NB (mm), as well as their changes and absolute values of changes before and after treatment, are represented as △U1-SN (°), △U1-NA (mm), △IMPA, △L1-NB(mm), |△IMPA|, |△L1-NB(mm)| (Note: △ = T2–T1).

### CBCT marking and measurement

**Fig. 4:**
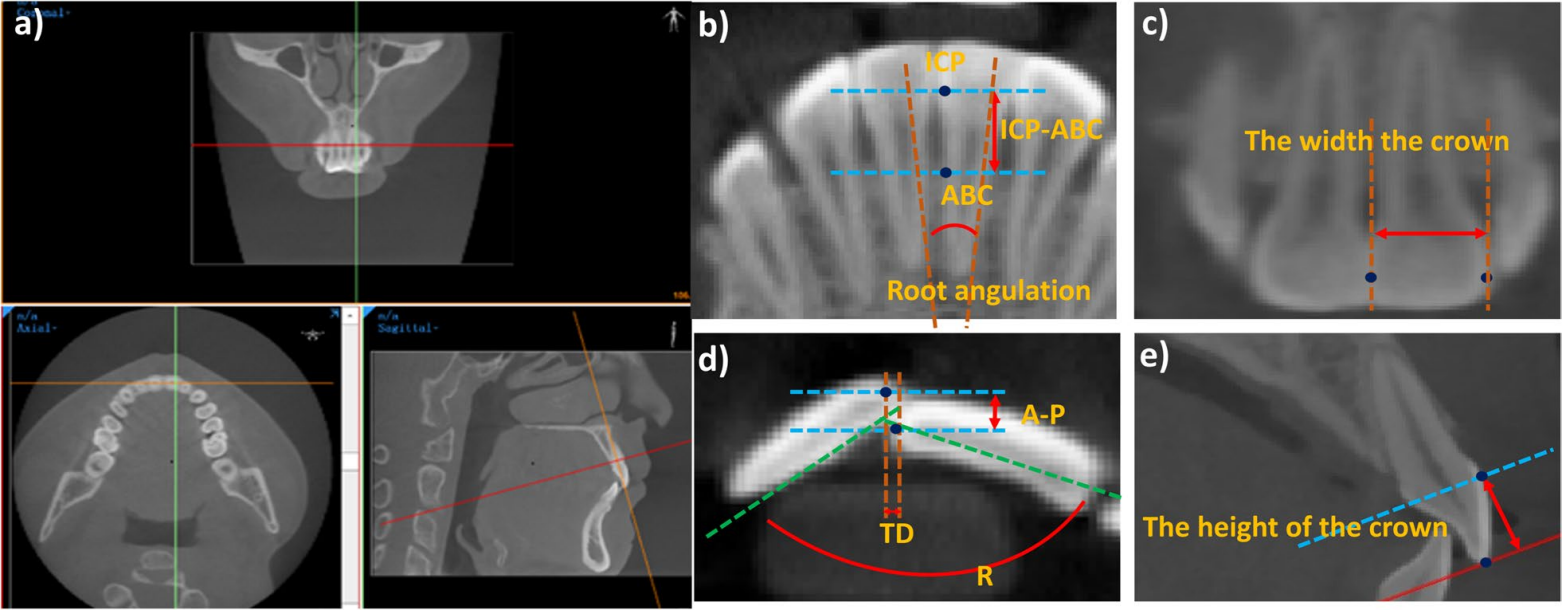
Adjustment and measurement of CBCT images. **a** On the sagittal plane, adjust the positions of the orange and red lines on the central incisor, and rotate the orange line to make it parallel to the long axis of the tooth. **b–c** On the coronal plane, adjust anteriorly and posteriorly until the pulp cavity of the central incisor is centered, to obtain the maximum coronal view of the central incisor. Locate ICP and ABC on this plane, and measure ICP-ABC, root angulation crown width. **d** Keeping the orange line parallel to the long axis of the tooth, adjust on the horizontal plane until the incisal edge is exposed, and measure A-P, TD, and R on this plane. **e** On the sagittal plane (where the red line is perpendicular to the long axis of the tooth), adjust until the pulp cavity of the central incisor is centered, and measure the crown height

All CBCT images were formatted into standard DICOM images and reconstructed into continuous slices of 0.3 mm in thickness. A dentist used the software Mimics Research 21.0 (Materialise NV, Leuven, Belgium) to perform axial adjustments on pre- and post-treatment CBCT scans and to conduct measurements. Position the central incisor in all three spatial planes. The method of orientation adjustment and measurement of the CBCT image is shown in Fig. 4. By manipulating the sagittal and coronal planes, an optimal view is achieved for measuring the desired parameters. Table 1 lists the names of the measurement items and landmarks.

**Table 1:** CBCT Measurement Landmarks and Items

Items	Definition
ICP	Interproximal contact point (Fig. 4b)
ABC	Alveolar bone crest (Fig. 4b)
ICP-ABC(mm)	The distance between the adjacent surface and the top of the alveolar bone crest (Fig. 4b)
Root Angulation (°)	The intersection Angle formed by the long axis of the central incisor tooth gathered the root towards the gingival side with positive value (Fig. 4b)
TD(mm)	Transverse Distances, Horizontal distance of the incisors (horizontal distance between the middle incisors), as shown in (Fig. 4d)
R(°)	The angle formed by the cutting margins of the two central incisors (Fig. 4d)
Crown width/height ratio	The ratio of width (Fig. 4c) to height (Fig. 4e) of the incisor teeth, in order to prevent the effect of the measurements of the two central incisors after treatment CBCT as the aspect ratio of the maxillary and mandibular incisor teeth

The variations in the distance from the contact point to the alveolar ridge crest, as well as the alterations in the angle formed by the incisal edges of the two central incisors, were calculated both before and after treatment. These changes are denoted as △ICP-ABC and △R (Note: △ represents the difference between T2 and T1).

### Sample size calculation, quality control and statistical analysis

The determination of the sample size in this study was calculated by a specific formula and guided by a previous study [24]. With a Z value of 1.96, which corresponds to a significance level of *P* = 0.05, and the expected prevalence of OGES being 39.6% as the medium value of the reported range between 35.4 and 43.7% [7–9], along with a d value of 0.06 [25], the calculations indicated that a minimum of 255 participants were required for the study.

A dentist randomly selected 20 lateral cephalometric radiographs and CBCTs, and continuously marked and measured them over a period of time, then re-measured them after 2 weeks. Intraclass correlation coefficient (ICC) was used to test the consistency of the results of the same measurement project in two occasions. After the repeatability and consistency of the measurements met the standard (ICC > 0.75), formal measurements began.

Statistical analysis was performed using SPSS 25.0 software (IBM Corp, Armonk, N.Y, USA), with a significance level set at *P* < 0.05. When comparing the differences in categorical variables such as gender, orthodontic treatment method, tooth extraction, sagittal and vertical skeletal patterns between two groups, Pearson’s chi-square test, continuity-corrected chi-square test, or Fisher’s exact probability method were used according to the type of contingency table and the minimum expected frequency. Continuous variables were tested for normality (Kolmogorov–Smirnov test or Shapiro–Wilk test) and homogeneity of variance. If they followed a normal distribution and had equal variance, the two-sample t-test was used. If they followed a normal distribution but had unequal variance, the two-sample t’ test was used. If they did not follow a normal distribution, the Mann–Whitney U test (also known as the Wilcoxon rank-sum test) was used. Variables that were statistically significant in the univariate analysis were extracted, and variables with multicollinearity (VIF > 10) were removed, followed by binary Logistic regression analysis.

## Results

### Consistency test results and characteristics of the study groups

Intraclass correlation coefficients (ICCs) from 0.897 to 0.990 (*P* < 0.01) confirmed high repeatability and consistency in the two measurements. Of the 330 patients, 130 were in the OGES group and 200 in the Non-OGES group. OGES was present in both maxillary and mandibular incisor areas in 32 (9.67%), only in the maxillary incisor area in 14 (4.23%), and only in the mandibular incisor area in 84 (25.38%). Demographic details and OGES distribution are in Table 2.

**Table 2:** Characteristics of the study groups

	OGES (n = 130)	Non-OGES (n = 200)
Male, n(%)	26 (20.00%)	73 (36.50%)
Female, n(%)	104 (80.00%)	127 (63.50%)
Age^#^	23.00 (15.00, 28.00)	14.00 (12.00, 19.00)
Treatment duration^#^	30.00 (23.75,39.00)	26.00 (20.25, 35.00)
Treatment Method, n(%)		
Fixed appliance	85 (65.38%)	146 (73.00%)
Invisalign	45 (34.62%)	54 (27.00%)

^#^: Non-normally distributed variables are expressed as median (25%, 75%)

### Overview and clinical characteristics of the study population

Based on the results of the chi-square test, we found a correlation between gender and the occurrence of orthodontic gingival recession (OGES) in the maxillary and mandibular central incisor regions (*P* < 0.05). Specifically, female patients exhibited a higher proportion of OGES. However, there was no significant correlation between the treatment methods, as well as tooth extraction, with the occurrence of OGES (*P* > 0.05) (Table 3).

**Table 3:** Correlation analysis of OGES and characteristics of two groups

Related Indicators		OGES	Non-OGES	Z/χ^2^/χ_c_^2^	*P*
*Maxilla*					
Age(y)^#^		25.00(17.75, 29.00)	14.00(12.00, 19.00)	Z = − 6.304	0.000**
Treatment duration (m)^#^		31.50(24.00, 40.00)	26.00(20.25, 35.00)	Z = − 2.467	0.014*
Gender	Male	5	73	χ_c_^2^ = 10.193	0.001**
	Female	41	127		
Treatment Method	Fixed	27	146	χ^2^ = 2.992	0.084
	Invisalign	18	54		
Tooth Extraction	Ye	20	76	χ^2^ = 0.406	0.524
	No	5	73		
*Mandible*					
Age(y)^#^		23.00(15.00, 28.00)	14.00(12.00, 19.00)	Z = − 7.392	0.000**
Treatment duration (m)^#^		30.00(23.25, 39.75)	26.00(20.25, 35.00)	Z = − 2.494	0.013*
Gender	Male	24	73	χ^2^ = 9.144	0.002**
	Female	94	127		
Treatment Method	Fixed	77	146	χ^2^ = 1.290	0.256
	Invisalign	38	54		
Tooth Extraction	Yes	51	66	χ^2^ = 3.786	0.052
	No	65	134		

^#^: Non-normally distributed variables were expressed as the median (25%, 75%), using the Mann–Whitney U test; χ^2^: The sample size was greater than 40, the minimum expected frequency was greater than 5, and Pearson's chi-square test was used; χ_c_^2^: For a 2 × 2 table, when the sample size is greater than 40 and the minimum expected frequency is less than 5, use the continuity-corrected chi-square test; *P < 0.05,**P < 0.01;

Meanwhile, we discovered a significant association between the initial age at diagnosis, treatment duration, and the occurrence of OGES in the maxillary and mandibular central incisors. The older the initial consultation age and the longer the treatment duration, the more likely OGES is to occur.

### Comparison of cephalometric measurements in the OGES and Non-OGES groups

Participants were classified by ANB and MP-SN (Table 4). Chi-square test analysis revealed no statistically significant association between sagittal/vertical skeletal pattern and the occurrence of OGES (*P* > 0.05).

**Table 4:** Correlation analysis of the skeletal pattern and OGES

Related Indicators		OGES	Non-OGES	χ2	*P*
*Maxilla*					
Sagittal skeletal pattern (ANB)	class I (2 ± 2.7°)	34	156	–	0.773
	class II (> 4.7°)	5	17		
	class III (< − 0.7°)	7	27		
Vertical skeletal pattern (MP-SN)	hyperdivergent (> 39°)	27	146	3.070	0.215
	normodivergent (35 ± 4°)	18	54		
	hypodivergent(< 31°)	26	122		
*Mandible*					
Sagittal skeletal pattern (ANB)	class I (2 ± 2.7°)	85	156	1.170	0.557
	class II (> 4.7°)	14	17		
	class III (< − 0.7°)	16	27		
Vertical skeletal pattern (MP-SN)	hyperdivergent (> 39°)	26	33	2.219	0.330
	normodivergent (35 ± 4°)	51	103		
	hypodivergent (< 31°)	37	64		

χ2: The sample size was greater than 40, the minimum expected frequency was greater than 5, and Pearson's chi-square test was used; –: 2xC list, with a minimum expected frequency of less than 5, using the Fisher exact probability method; *P < 0.05, **P < 0.01

U1-SN(T1), U1-SN(T2), U1-NA distance(T2), ΔU1-SN, ΔU1-NA correlated significantly with OGES in maxillary central incisors (*P* < 0.05), with smaller values indicating higher OGES likelihood. Additionally, a smaller ΔL1-NB distance indicated a greater possibility of OGES. No statistically significant differences were found for the remaining measurement indicators (as shown in Table 5).

**Table 5:** Correlation analysis of OGES and cephalometric measurements

Related Indicators	OGES	Non-OGES	Z/t	*P*
*Maxilla*				
ANB^#^	3.82(2.22, 4.97)	3.04(1.74, 4.36)	Z = − 1.435	0.151
MP-SN^##^	35.02 ± 6.35	33.84 ± 5.70	t = 1.237	0.217
U1-SN(T1)^##^	103.12 ± 9.28	106.28 ± 8.03	t = − 2.337	0.020*
U1-NA(mm,T1)^#^	5.53 ± 3.27	6.06 ± 2.78	t = 1.119	0.263
U1-SN(T2)^##^	99.07 ± 8.15	105.31 ± 8.05	t = − 4.724	0.000**
U1-NA(mm,T2)^##^	4.10 ± 2.82	5.54 ± 2.88	t = 3.157	0.002**
△U1-SN^#^	− 4.24(− 8.51, 0.14)	− 0.82(− 4.36, 3.47)	Z = − 3.067	0.002**
△U1-NA(mm)^#^	− 0.75(− 3.38, 0.15)	− 0.27(− 1.95, 1.34)	Z = − 2.026	0.043*
|△U1-SN|^#^	5.05(2.09, 9.25)	3.96(2.16, 7.63)	Z = − 1.216	0.224
|△U1-NA(mm)|^#^	2.25(0.45, 3.84)	1.62(0.60, 3.05)	Z = − 0.777	0.437
*Mandible*				
ANB^#^	3.57(1.48, 4.70)	3.04(1.74, 4.35)	Z = 0.953	0.341
MP-SN^##^	34.39 ± 6.07	33.84 ± 5.70	t = − 0.818	0.414
IMPA(T1)^##^	96.05 ± 8.14	95.54 ± 6.61	t = − 0.602	0.548
L1-NB(mm,T1)^##^	5.99 ± 2.59	5.57 ± 2.18	t = − 1.556	0.121
IMPA(T2) ^##^	96.27 ± 7.80	97.05 ± 7.98	t = 0.842	0.401
L1-NB(mm,T2) ^##^	5.47 ± 1.85	5.70 ± 2.16	t = 0.961	0.337
△IMPA^#^	0.07(− 5.14, 5.28)	0.38(− 2.25, 6.09)	Z = − 1.299	0.194
△L1-NB(mm)^#^	− 0.06(− 1.89, 1.03)	0.16(− 1.18, 1.38)	Z = − 1.983	0.047*
|△IMPA|^#^	5.21(2.12, 8.79)	3.86(1.74, 7.80)	Z = − 1.465	0.143
|△L1-NB|^#^	1.37(0.61, 2.52)	1.27(0.54, 2.36)	Z = 0.973	0.331

^#^: Non-normally distributed variables, expressed as the median (25%, 75%), using the Mann–Whitney U test; ^##^: Normally distributed variables, expressed as mean and standard deviation (*X* ± SD), if equal variance, using two-sample t-test, if uneven variance, using two-sample t-test; *P < 0.05, **P < 0.01

### Comparison of CBCT between the OGES and Non-OGES groups

After measuring and analyzing the CBCT data of the OGES group and the Non-OGES Group, we found that in the maxillary central incisor region, the distances between ABC and ICP, △ICP-ABC, and A-P overlap before and after treatment in the OGES group were significantly greater than those in the Non-OGES Group (*P* < 0.05). The remaining measurement indicators, including TD, △R, root angulation, and crown width-to-height ratio, showed no significant correlation (Table 6). In the mandibular central incisor region, the distances between ABC and ICP, and △ICP-ABC before and after treatment in the OGES group were also significantly greater than those in the Non-OGES Group (*P* < 0.05).

**Table 6:** Correlation analysis of OGES and CBCT indicators

Related Indicators	OGES	Non-OGES	Z/t/t'	P
*Maxilla*				
ICP-ABC(T1)^##^	6.13 ± 1.53	5.37 ± 0.77	t' = − 2.562	0.006**
ICP-ABC(T2)^##^	6.63 ± 1.05	5.20 ± 0.56	t' = − 5.543	0.000**
△ICP-ABC^##^	0.51 ± 0.55	0.19 ± 0.49	t = 0.612	0.034
A–P^#^	0.47(0.07, 0.79)	0.00(0.00, 0.55)	Z = − 2.465	0.014*
TD^#^	0.00(0.00, 0.57)	0.00(0.00, 0.41)	Z = − 1.242	0.214
△R^#^	− 1.92(− 26.78, 3.60)	0.62(− 9.05, 2.07)	Z = − 0.599	0.549
Root angulation^##^	2.06 ± 3.38	1.99 ± 3.63	t = − 0.061	0.952
The width and height ratio of the crown	0.73(0.71, 0.75)	0.72(0.71, 0.77)	Z = − 0.473	0.141
*Mandible*				
ICP-ABC(T1)^##^	5.72 ± 0.74	4.80 ± 0.72	t = − 5.068	0.000**
ICP-ABC(T2)^#^	6.23(5.96, 7.16)	5.21(4.63, 5.55)	Z = − 5.932	0.000**
△ICP-ABC^##^	0.79 ± 0.68	0.37 ± 0.40	t' = − 3.086	0.033*
A-P^#^	0.80(0.00, 2.03)	0.83(0.00, 1.85)	Z = − 1.441	0.150
TD^#^	0.00(0.00, 0.23)	0.00(0.00, 0.28)	Z = − 0.439	0.661
△R^#^	− 2.12(− 26.98, 1.69)	− 1.20(− 25.50, 1.27)	Z = − 0.353	0.724
Root angulation^#^	2.45(1.34, 5.23)	3.87(− 0.32, 4.67)	Z = − 0.981	0.327
The width and height ratio of the crown^##^	0.62 ± 0.03	0.63 ± 0.04	t = 0.851	0.398

^#^: Non-normally distributed variables, expressed as the median (25%, 75%), using the Mann–Whitney U test; ^##^: Normally distributed variables, expressed as mean and standard deviation (*X* ± SD), if equal variance, using two-sample t-test, if uneven variance, using two-sample t-test; *P < 0.05, **P < 0.01

### Multivariate analysis of correlated indicators

Due to differences and sample size limitations in CBCT measurements compared to the study population and lateral skull X-ray film measurements, this experiment focuses on multivariate analysis of demographic, clinical characteristics, and cephalometric indicators. Binary logistic regression identified initial consultation age, treatment duration, and △U1-SN as significant factors affecting OGES occurrence in the maxillary central incisor area, with older age, longer treatment, and smaller △U1-SN correlating with greater retraction and higher OGES likelihood (Table 7). Similarly, initial consultation age and treatment duration were significantly associated with OGES in the mandibular central incisor area.

**Table 7:** Multivariate analysis of OGES in the maxillary central incisor area

Related Indicators	B	SE	Waldχ^2^	P	OR	95% CI
*Maxilla*						
Gender	− 0.713	0.555	1.651	0.199	0.490	0.165–1.455
Age	0.146	0.029	25.907	0.000**	1.157	1.094–1.224
Treatment duration	0.035	0.017	4.188	0.041*	1.036	1.001–1.071
U1-SN (T1)	− 0.050	0.028	3.228	0.072	0.951	0.901–1.005
△U1-SN(°)	− 0.074	0.033	4.952	0.026*	0.929	0.870–0.991
*Mandible*						
Gender	− 0.317	0.312	1.035	0.309	0.728	0.395–1.342
Age	0.128	0.020	39.425	0.000**	1.137	1.092–1.183
Treatment duration	0.026	0.011	5.518	0.019*	1.027	1.004–1.050
△L1-NB (mm)	− 0.090	0.065	1.905	0.168	0.914	0.805–1.038

B: Beta Coefficient; SE: Standard Error; Waldχ^2^: Wald Chi-squared test; OR: odds ratio; *P < 0.05, **P < 0.01; CI: Confidence Interval.

## Discussion

Previous studies have reported risk factors including gender [10, 11], age [8, 10, 12–14], gingival biotype [15], oral hygiene status [16], treatment duration [17], tooth extraction [18–21], and crowding [8, 22]. However, there is still controversy surrounding the perspectives.

In our analysis, females are more prone to OGES, which may be associated with gender differences in gingival biology and hormonal levels [20]. However, is not consistent across all research [10, 11] and may be influenced by the sample size and the interplay of multiple factors. Age also emerged as a significant factor, with an increase in the initial diagnosis age of patients correlating to a higher risk of OGES, aligning with the majority of existing research [8, 10, 12–14]. Furthermore, the duration of treatment is significantly correlated with the occurrence of OGES, consistent with previous research findings [26]. The sagittal movement of anterior teeth, especially the lingual movement of the maxillary central incisor, was found to significantly correlate with OGES occurrence and supported by multivariate analysis. In our study, we found no evidence to suggest that labial movement of anterior teeth leads to the occurrence of OGES, which is consistent with the findings of Vasconcelos et al. [17] and An et al. [11]. The inclination of the anterior teeth was found to have a correlation with OGES, with a more lingually inclined position of both the initial and final angles of the maxillary central incisors correlating to a higher likelihood of OGES. In our study, the mean value of U1-SN after treatment for the non-OGES group of maxillary central incisors was 105.31 degrees, which was significantly greater than that of the OGES group at 99.07 degrees. According to Andrews’ Six Elements, teeth that are upright in the center of the alveolar bone are crucial for achieving long-term stable treatment outcomes [27]. In contrast to the maxillary central incisors, we observed no statistically significant correlation between the lingual inclination angle of the mandibular central incisors and the occurrence of OGES. This finding might be attributed to the thinner gingival tissue surrounding the mandibular central incisors, which could predispose them to a higher incidence of OGES. Currently, there is a scarcity of research examining the link between alveolar bone height and OGES using CBCT. In our study, we discovered that the distance from the contact point of the maxillary central incisor to the alveolar ridge crest was greater in patients who experienced OGES. This suggests a potential association between alveolar bone height and the likelihood of OGES, a finding that echoes previous research conclusions [11, 28] and confirmed the research by Tarnow et al. [29]. The relationship between tooth crowding and the development of OGES is a matter of debate [8, 22]. We assessed the degree of crowding by measuring specific distances and angles of the central incisors, finding a significant association only with the A-P distance of the upper central incisors and OGES occurrence in univariate analysis.

Additionally, other factors such as treatment method, extraction of teeth, sagittal and vertical skeletal patterns, root angulation, and crown morphology were compared, but no significant statistical differences were found between the groups. This is consistent with some studies [18, 26, 30] but not with others [3, 22, 31]. We speculate that differences in the number and characteristics of the patients included may account for the divergence in conclusions between our study and previous ones.

## Limitation

It is acknowledged that there are inherent limitations within the scope of this study. Initially, a number of factors were not taken into account, such as gingival biotype, oral hygiene status, and the practice of interproximal reduction (IPR). Furthermore, the sample size of CBCT scans was constrained, as these were not deemed a clinical necessity for every patient evaluated. In the pursuit of further research, it is recommended to incorporate sophisticated methodologies, such as machine learning, on a larger sample size. Additionally, the conduct of prospective studies may be warranted to enhance our comprehension of the risk factors associated with OGES.

## Conclusion

In conclusion, our study’s univariate analysis indicated that the occurrence of OGES in the upper central incisors is significantly associated with initial consultation age, treatment duration, initial and final angular positions and changes of the anterior teeth, and alveolar bone height. For the lower central incisors, similar factors were identified. Binary Logistic regression analysis confirmed that initial consultation age and treatment duration are independent influencing factors for the occurrence of OGES. Therefore, in orthodontic treatment, it is crucial to consider these various factors comprehensively to prevent or reduce the occurrence of OGES, ensuring a balance between facial aesthetics and smile aesthetics.

## Data Availability

No datasets were generated or analysed during the current study.

## Abbreviations

A: Subspinale
ABC: Alveolar bone crest
A–P: Anterior–posterior distance
B: Suprakental
CBCT: Cone beam computed tomography
Gn: Gnathion
Go: Gonion
ICC: Intraclass correlation coefficient
ICP: Interproximal contact point
ICP-ABC: The distance between the adjacent surface and the top of the alveolar bone crest
LI: Lower incisor
LIA: Lower incisor apex
MP: Mandibular plane
N: Nasal
OGES: Open gingival embrasure space
S: Sella
SN: Anterior cranial base plane
TD: Transverse distances, horizontal distance of the incisors (horizontal distance between the middle incisors)
TD: Tansverse distances
UI: Upper incisor
UIA: Upper incisor apex
